# Habitat heterogeneity drives the geographical distribution of beta diversity: the case of New Zealand stream invertebrates

**DOI:** 10.1002/ece3.1124

**Published:** 2014-06-02

**Authors:** Anna Astorga, Russell Death, Fiona Death, Riku Paavola, Manas Chakraborty, Timo Muotka

**Affiliations:** 1 Department of Biology, University of OuluP.O. Box 3000, FI-90014, Oulu, Finland; 2 Institute of Agriculture and Environment - Ecology, Massey University, Private Bag11-222, Palmerston North, New Zealand; 3 Finnish Environment Institute, Natural Environment Centre, University of OuluP.O. Box 413, FI-90014, Oulu, Finland; 4 Thule Institute, Oulanka Research StationOulanka, Finland; 5 Horizons Regional Council, Private Bag11025, Palmerston North, New Zealand

**Keywords:** Beta diversity, habitat heterogeneity, macroinvertebrates, null models, productivity, streams

## Abstract

To define whether the beta diversity of stream invertebrate communities in New Zealand exhibits geographical variation unexplained by variation in gamma diversity and, if so, what mechanisms (productivity, habitat heterogeneity, dispersal limitation, disturbance) best explain the observed broad-scale beta diversity patterns. We sampled 120 streams across eight regions (stream catchments), spanning a north–south gradient of 12° of latitude, and calculated beta diversity (with both species richness and abundance data) for each region. We explored through a null model if beta diversity deviates from the expectation of stochastic assembly processes and whether the magnitude of the deviation varies geographically. We then performed multimodel inference analysis on the key environmental drivers of beta diversity, using Akaike's information criterion and model and predictor weights to select the best model(s) explaining beta diversity. Beta diversity was, unexpectedly, highest in the South Island. The null model analysis revealed that beta diversity was greater than expected by chance in all eight regions, but the magnitude of beta deviation was higher in the South Island, suggesting differences in environmental filtering and/or dispersal limitation between North and South Island. Habitat heterogeneity was the predominant driver of beta diversity of stream macroinvertebrates, with productivity having a secondary, and negative, contribution. This is one of the first studies accounting for stochastic effects while examining the ecological drivers of beta diversity. Our results suggest that local environmental heterogeneity may be the strongest determinant of beta diversity of stream invertebrates, more so than regional- or landscape-scale variables.

## Introduction

Beta diversity is central to a wide array of theoretical and applied ecological questions, including scaling of diversity, delineation of biotic regions, and conservation of biodiversity. It is a measure of compositional difference across space, higher beta diversity meaning higher level of compositional variation, and it provides a link between diversity at local scales (alpha diversity) and the regional species pool (gamma diversity) (Koleff et al. 2003). Beta diversity exhibits a latitudinal gradient, increasing toward the equator in several organism groups including, for example, plants, birds, mammals, and freshwater fish (e.g., Qian and Ricklefs 2007 Qian et al. 2009 Leprieur et al. 2011). This pattern is scale-dependent, with a stronger latitudinal gradient for gamma than alpha diversity (Hillebrand 2004). However, as pointed out by Hawkins and Diniz-Filho (2004), it is not latitude per se that determines species diversity, but latitude is rather associated with spatially patterned environmental variables that eventually regulate biotic patterns.

The main drivers of beta diversity can be roughly divided into three groups: (1) dispersal limitation, which is related to species traits, spatial arrangement of communities, and site history; (2) environmental heterogeneity that generates niches where some species are favored over others; and (3) productivity whereby more productive areas support higher regional diversity (“species-energy hypothesis”; Currie 1991). Often these factors are intertwined in complex ways; for example, productivity may cause higher regional diversity of particularly rare species (Currie et al. 2004), but it may as well be correlated with other factors (e.g., environmental heterogeneity) that generate diversity (Morin 2000). Dispersal limitation and historical contingencies (e.g., glaciations) have been proposed to explain continental-scale beta diversity patterns of plants, fish and amphibians, especially at higher latitudes (Qian and Ricklefs 2007; Leprieur et al. 2011; Baselga et al. 2012), while environmental processes often explain beta diversity at regional scales and lower latitudes (Qian and Ricklefs 2007).

Productivity is often linked to beta diversity, with higher beta diversity at higher productivities resulting from high regional diversity and increased niche specialization (Harrison et al. 2011). Alternatively, Chase (2010) found stochastic processes due to differential colonization history and priority effects to be more important in systems with higher productivity. Habitat heterogeneity has also been identified as a key driver of beta diversity, with fragmented landscapes showing higher spatial variability in their biota than more homogeneous regions (Veech and Crist 2007). Aside from the increase in niche availability, topographically heterogeneous landscapes may influence beta diversity through dispersal limitation (Finn et al. 2006). Nevertheless, given that beta diversity is a simple function of alpha and gamma diversity, caution must be taken before ascribing any ecological mechanisms to differences in beta diversity between regions. Because gamma diversity also varies with latitude, the influence of gamma diversity on beta diversity must be accounted for if ecological explanations to variation in beta diversity are to be offered (Kraft et al. 2011).

Freshwater habitats are islands within a terrestrial continuum; thus, both dispersal limitation and environmental processes may play important roles in shaping beta diversity patterns of freshwater communities. For species with strictly aquatic dispersal, such as freshwater fish, drainage basins are separated by impassable barriers. The diversity of freshwater invertebrates with potential for aerial dispersal frequently shows a strong relationship with local environmental heterogeneity, hydrological variability, and regional topography (Death and Joy 2004; Astorga et al. 2011). Dispersal ability (or, rather, dispersal limitation) may also be important, however. For example, Finn et al. (2006) found that macroinvertebrate populations in isolated mountain streams with deep valleys often show unique genetic structure. Furthermore, macroinvertebrate taxa with poor dispersal ability have higher species turnover rates than do good dispersers (Thompson and Townsend 2006; Astorga et al. 2012). Benthic macroinvertebrate communities in New Zealand streams are relatively species poor, characterized by a high degree of generalism in habitat and food requirements (Collier and Winterbourn 2000). New Zealand streams are also flashy, with communities shaped by flow-related disturbances (Death 2002). Furthermore, stream catchments, particularly in the South Island, are topographically variable (Collier and Winterbourn 2000), resulting in high habitat heterogeneity and potentially limited among-stream dispersal.

We explored the geographic patterns of beta diversity of New Zealand stream invertebrates to (1) determine whether beta diversity exhibits a latitudinal pattern; and (2) explore whether one or more of the ecological mechanisms proposed to explain patterns in beta diversity might also provide explanation for the beta diversity of stream invertebrate communities. We stratified our sampling by habitat type, focusing on near-pristine forest streams across a more than 1300-km north-to-south gradient, encompassing twelve of the lotic ecoregions of New Zealand (see Harding and Winterbourn 1997). Acknowledging that latitude per se cannot be a determinant of diversity but only a correlate of causal environmental factors (e.g., Gaston 2000), we predicted that beta diversity of macroinvertebrates is higher in regions with higher primary productivity, resulting in lower beta diversity toward higher latitudes. Additionally, beta diversity could be positively related to habitat heterogeneity (environmental heterogeneity hypothesis) and/or to catchment steepness (dispersal limitation hypothesis). Lastly, given the flashy nature of New Zealand streams, we hypothesized that variability in the intensity of flow-related disturbances could increase beta diversity by affecting local extinction/colonization dynamics; thus, sites within a region may be in different stages of colonization following disturbance (intensity of disturbance hypothesis).

## Methods

### Study sites

We sampled in total 120 streams: 15 stream sites in each of eight regions in New Zealand, spanning a north–south latitudinal gradient of 12° (Appendix S1). The regions were (from north to south): Northland (NL), Urewera (UR), Egmont (EG), Tararua (TA), Kahurangi (KA), Arthurs Pass (AP), West Coast (WE), and Fiordland (FI). Within each region, streams were sampled mainly from within National or State Forest Parks so that sites had no urban areas in their upstream catchments, a maximum of 14% exotic forest plantations, and maximum of 30% pasture land (surrogate for agriculture). However, these values were an exception as less than 12% of sites had between 5–12% of pasture land in their catchment and all sites had always at least a 50-meter riparian forest buffer (Freshwater Ecosystems of New Zealand (FENZ) (Leathwick et al. 2010). To restrict our analyses to a single habitat type, we focused on riffles in forest streams (less than 7 m wide). Streams were also selected based on their accessibility.

### Biotic sampling

Sampling was conducted in the Austral summer/autumn between February and April 2006. Benthic macroinvertebrates were sampled by taking a two-minute kick-net sample (net mesh size 0.3 mm) at each site, aiming to cover most microhabitats present in a riffle section of ca. 100 m^2^. Samples of this size cover 1.3 m^2^ of the stream bed and capture about 75% of the benthic invertebrate species present in a riffle, missing mainly species that occur sporadically in running waters (Mykrä et al. 2006). Macroinvertebrates and associated material were preserved in 70% alcohol and later sorted in the laboratory. All individuals were counted and identified to the lowest feasible taxonomic level, usually genus or species, using the keys of Winterbourn et al. (2000). Our analysis includes all major groups of macroinvertebrates, but as chironomid midges were only identified to tribe level, they were excluded from all analyses.

### Environmental variables

Several in-stream and riparian characteristics were measured at each study site (Appendix S2). Depth and current velocity (at 0.4 × depth) were measured with a Flow-mate (Marsh-McBirney 2000) at 40 random locations in cross-channel transects. Percentage of macrophytes in the wetted channel was visually estimated for each reach. Mean stream width was recorded, and channel slope was measured with an Abney level over 10–20 m, depending on channel steepness. Substrate composition was determined by counting the number of stones in each of 13 size classes (bedrock, >300, 300–128, 128–90.5, 90.5–64, 64–45.3, 45.3–32, 32–22.6, 22.6–16, 16–11.3, 11.3–8 8–5, <5 mm) for 100 particles collected at 1-m intervals along a path at 45° to each stream bank. These measures were converted to a single substrate size index (SI) by summing the mid-point values of each size class weighted by the number of stones in each class (bedrock was assigned a nominal size of 400 mm). Canopy cover was measured at 20 locations in evenly spaced cross-channel transects. Stream bed stability was assessed with the bottom component of the Pfankuch Stability Index (Death and Winterbourn 1995). It involves allocation of an observer's subjective evaluation of six wetted channel attributes (substrate brightness, angularity, consolidation, percentage of stable materials, scouring, and amount of clinging aquatic vegetation) to four predetermined categories with weighted scores. The sum of the scores results in a bottom stability index where high values represent low stability. Finally, five stones were randomly collected from each site and frozen for later analysis of periphyton biomass, measured as chl-a (μg·cm^−2^). In the laboratory, pigments were extracted by soaking the stones in 90% acetone for 24 h at 5°C in the dark. Absorbance readings were taken using a Cary 50™ Conc UV-Visible spectrophotometer, and chlorophyll-a was calculated using the method of Steinman and Lamberti (1996). Corrections were made for stone surface area (Graham et al. 1988), and assuming only the top half of the stone was exposed to light and thus suitable for periphyton growth. Upstream catchment topography and land use were obtained from Freshwater Ecosystems of New Zealand (FENZ) (Leathwick et al. 2010) and the New Zealand River Environment Classification (REC) derived from a 30 m Digital Elevation Model (Snelder and Biggs 2002) (see Appendix S2 for a list of environmental variables used in our analysis).

### Data analysis

We explored beta diversity patterns in two different ways that characterize different aspects of community structure. First, beta diversity was calculated as the multiplicative beta partition (*β* = *γ/α*, Whittaker 1960; hereafter referred to as beta_*w*_). Most diversity indices do not measure diversity per se and must be converted to number equivalents (“true diversities”) (Jost 2007). We used both species richness (which is its own numbers equivalent) and Shannon entropy (H') which was converted to numbers equivalents by taking its exponent (expH') (“effective diversity”, Jost 2007). We define alpha diversity as the average species richness (or effective diversity) of a single stream, gamma diversity as the total richness (or total effective diversity) of the 15 streams in a region, and beta_*w*_ (*β*_*w*)_ diversity as the regional-to-local diversity ratio for each region (how many times regional richness exceeds that of its constituent sites on average). The patterns of *β*_*w*_ diversity based on species-richness data place considerable emphasis on rare species, while effective *β*_*w*_ diversity emphasizes differences in relative abundances of species, with numerically dominant species playing a stronger role (Jost 2007). To gain greater insight into the distribution patterns of macroinvertebrate species, we explored the occupancy–frequency plots separately for each island. We tested for the modality of the distributions using the method of Tokeshi (1992). This analysis tests whether the occupancy–frequency distribution is significantly (*P* < 0.05) right-skewed (most species are rare), significantly left-skewed (most species are common) or significantly bimodal, under the null hypothesis of a uniform distribution.

Kraft et al. (2011) showed that variation in beta diversity across broad geographic gradients is more likely driven by gamma diversity than by local community assembly processes. Thus, before inferring that ecological processes drive the observed beta diversity differences between our study regions, we applied a null model approach to assess whether beta diversity deviates from the expectation of stochastic assembly processes and whether the magnitude of the deviation varies geographically. We compared the observed *β*_*w*_ diversity to patterns generated by a null model using the approach of Kraft et al. (2011). The null model randomly shuffles individuals among sites while preserving gamma diversity, the relative abundance of species in a region, and the number of individuals per site. This corrects for gamma dependency and provides expected values of *β*_*w*_ diversity for each site based on random sampling from the species pool. We calculated *β*_*w*_ deviation as the observed *β*_*w*_ diversity minus the mean of the null distribution of *β*_*w*_ values, divided by the standard deviation of this distribution (see Kraft et al. 2011).

Second, we calculated beta diversity as multivariate dispersion (hereafter, *β*_*d*_) based on Sørensen's dissimilarity measure, separately for each of the eight regions. As Whittaker's (1960) index is calculated as a single value for a given region, it does not allow statistical comparison of beta diversity between regions. Multivariate dispersion has proved to be a robust and powerful approach to measuring beta diversity, and it also provides a test for differences in beta diversity among regions (Anderson et al. 2006). We measured *β*_*d*_ diversity for a region as the average distance (or dissimilarity) from an individual site to the region centroid and tested the null hypothesis of no differences in beta diversity among regions. The test of homogeneity of multivariate dispersions calculates an *F*-statistic to compare the average distance of a site to the region centroid, in the space defined by the chosen dissimilarity measure (an analog to Levene's test). A *P*-value is then obtained by permuting least-square residuals (Anderson 2006). For comparative purpose, we used the same protocol to compare the multivariate dispersion of New Zealand's North and South Island macroinvertebrate communities as the average distance from an individual site to the respective island centroid.

To explore the relationship between beta diversity and environmental variables, we selected factors known to be important regulators of stream macroinvertebrate diversity at local to regional extents (e.g., Death and Joy 2004; Astorga et al. 2011): productivity, disturbance, dispersal, and habitat heterogeneity. As the surrogate of productivity, we used chlorophyll-a concentration, which is strongly linked with stream primary productivity (Morin 1997). We used the bottom component of the Pfankuch index to measure disturbance at a scale relevant for benthic invertebrates (Death and Winterbourn 1995). As a surrogate for dispersal limitation imposed on organisms by landscape structure, we used catchment topographic steepness (percentage of catchment area with slope >30°). We measured habitat heterogeneity by calculating the multivariate dispersion (Euclidean distance) of each site from its regional centroid (Anderson 2006) based on six in-stream variables: current velocity, slope, depth, canopy cover, substratum size, and macrophyte cover. Compared to topographic heterogeneity (which has been frequently used as a broad-scale indicator of habitat heterogeneity in terrestrial studies), our measure should be a more effective indicator of habitat diversity because it operates at a scale where most stream organisms perceive heterogeneity.

We first examined the relationship of each of the four environmental factors (chlorophyll-a concentration, bottom Pfankuch, catchment steepness, and habitat heterogeneity) to *β*_*d*_ diversity (Sørensen-based multivariate distance of each site to the corresponding regional centroid) in simple linear regressions; relationship of beta_*w*_ to environmental variables could not be tested because this index was calculated at a different scale. We then included each of these factors into a multiple regression model and performed multimodel inference analysis using Akaike's information criterion (*AICc* corrected for small sample size). Model and predictor weights were used to select the best model (Burnham and Anderson 2004). *AICc* allows one to compare and rank multiple competing models and to estimate which of them best approximates the processes underlying a biological pattern. Similarly, predictor weights give an idea of the importance of a variable: if a predictor appears in all of the top models, then the summed Akaike weight will tend toward one (Burnham and Anderson 2004). Finally, model weights estimate the probability that a particular model is the best one given the candidate set of models considered (Burnham and Anderson 2004). We used the package MuMIn in R program for the multimodel inference analysis (Barton 2012).

## Results

**Figure 1:**
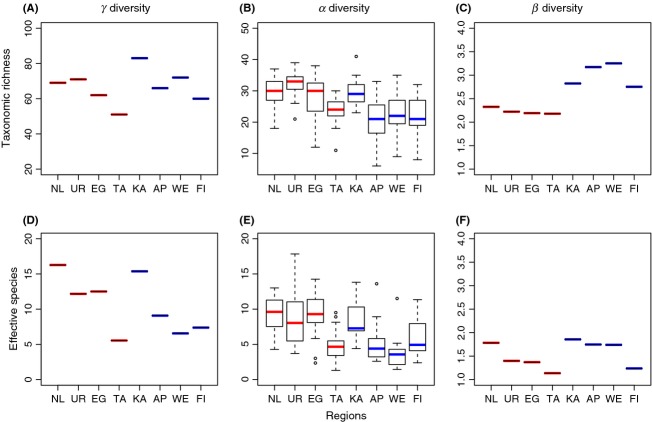
Gamma (*γ*), alpha (*α*), and beta (*β_w_*) diversity of stream macroinvertebrates across eight regions (catchments) in New Zealand, separately for species-richness-based data (A–C) and for effective diversity (exponential of Shannon diversity) (D–F). Regions are presented in north-to-south order (NL, Northland; UR, Urewera; EG, Egmont; TA, Tararua; KA, Kahurangi; AP, Arthur's Pass; WE, Westland; and FI, Fiordland). Red lines: North Island regions, blue lines: South Island regions. Plots represent total richness (or total effective diversity) across the 15 streams in a region (gamma diversity), median species richness (or median effective diversity) for a region and the regional-to-local diversity ratio for a region (*β*_*w*_ = *γ*/*α*).

**Figure 2:**
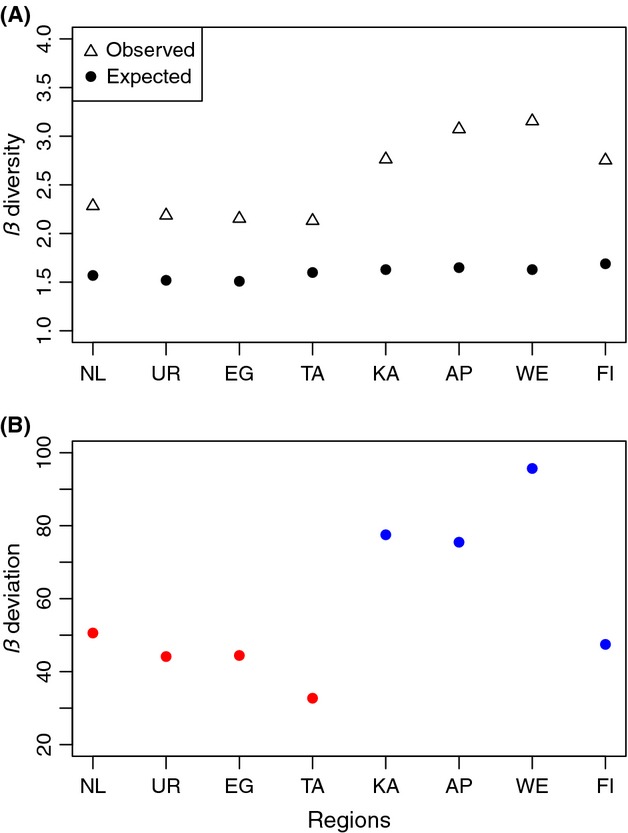
(A) Patterns in observed and expected multiplicative *β*_*w*_ diversity of stream macroinvertebrate communities in eight regions of New Zealand, organized along a latitudinal gradient from lower to higher latitudes. (B) Patterns in *β*_*w*_ deviation across the eight regions. Red symbols: North Island regions, blue symbols: South Island regions. *β*_*w*_ deviation represents a standard effect size for the deviation of beta diversity from a null model that corrects for dependence of beta on gamma diversity (see text for further details). Positive values indicate higher *β*_*w*_ diversity than expected by chance.

Gamma diversity of benthic macroinvertebrates decreased from north to south on each island (Fig. 1A). Alpha diversity exhibited an overall decreasing trend toward the south (Fig. 1B), while *β*_*w*_ diversity was highest in the South Island (Fig. 1C). Geographical patterns for effective diversity (expH') were somewhat different (Fig. 1D–F). No overall north-to-south pattern was evident: instead, effective beta diversity was highest in the northernmost region in both islands then decreased toward the south. The null model analysis revealed that *β*_*w*_ diversity was greater than expected by chance in all regions (Fig. 2A). The magnitude of beta deviation was higher in the South Island (Fig. 2B).

**Figure 3:**
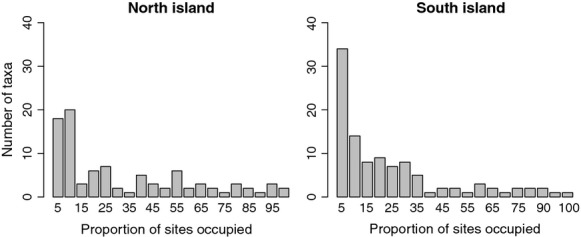
Occupancy–frequency distributions of stream macroinvertebrate communities in the North and South Island of New Zealand. The proportion of sites occupied is divided into occupancy–frequency classes (i.e., the first class represents 0.2–5% of sites occupied, the second one represents 5–10% sites occupied).

The occupancy–frequency distributions of stream invertebrates were significantly right-skewed in both islands, although more strongly so in the South Island (North Island *n* = 92 sites, *P* = 0.004; South Island *n* = 105, *P* < 0.0001). Accordingly, the South Island exhibited a higher number of low-occupancy species than the North Island (for example, species present in less than 20% of sites: 65 vs. 47 taxa, respectively) (Fig. 3). Taxa that exhibited high occupancies (>80% of sites; e.g., *Deleatidium* and *Nesameletus* mayflies, the *Zelandoperla* stonefly, the caddis larva *Psilochorema*, and the cranefly *Aphrophila*) were equally common on both islands.

**Figure 4:**
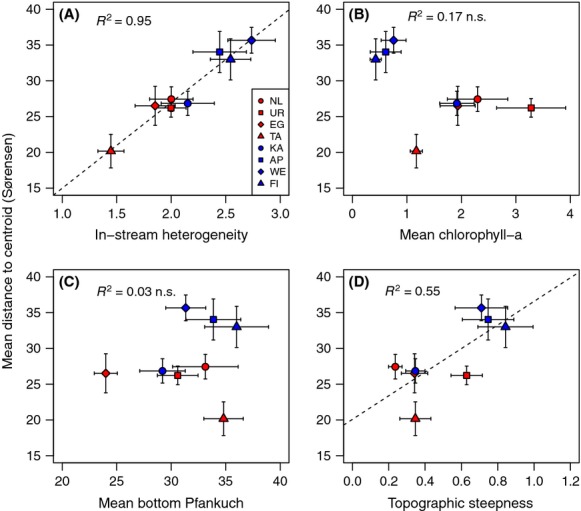
Beta diversity–environment relationships for the New Zealand stream macroinvertebrate communities. Red symbols: North Island regions, blue symbols: South Island regions mean (±1 SE) distance of a site (*n* = 15 sites per region) to the respective group (region) centroid in relation to mean (±1 SE) for habitat heterogeneity (Euclidean distance) (*y* = 2.9 + 11.9*x, P* < 0.0001) (A), chlorophyll-a concentration (μg·cm^−2^) (B), bottom Pfankuch (stream disturbance index) (C) and topographic steepness (D).

Beta diversity based on multivariate dispersion (*β*_*d*_) was significantly different among the eight regions (region effect, *F* = 5.19, df = 7, *P* = 0.002) and also greater in the South Island than the North Island (Island effect, *F* = 20.29, df = 58, *P* = 0.001). In linear regressions, *β*_*d*_ diversity increased significantly, and strongly so, with habitat heterogeneity (Fig. 4A), whereas all other univariate relationships were nonsignificant (Fig. 4B–D). However, the relationship between beta diversity and productivity (chlorophyll-a) was strongly affected by one North Island region, the Tararua (see Fig. 4B), and removing this region produced a significantly negative pattern (adjusted *r*^2^ = 0.72, *P* = 0.009).

The best multiple linear model included habitat heterogeneity and chlorophyll-a with Akaike weight of 0.35. Habitat heterogeneity had a positive regression coefficient, while chlorophyll-a was negatively related to beta diversity. The second best model included only habitat heterogeneity and did not differ appreciably from the best model (delta *AICc*: 0.98). Habitat heterogeneity appeared in all four top-ranking models (delta *AICc* < 2.0; see Appendix S3). The explained variance of the selected models varied between 20–25%, so not particularly high, yet very common for a survey type of data sampled in streams across such a large spatial extent. When the relative importance of each predictor was examined by summing the Akaike weights for each model where that variable appeared, habitat heterogeneity had a weight of 0.99, that is, it was included in 99% of all possible models. Chlorophyll-a had a weight of 0.57, while the other variables had a weight of below 0.35.

## Discussion

We compared beta diversity of stream invertebrate communities in eight regions across New Zealand and found that, contrary to our expectations, beta diversity based on richness data (Whittaker's multiplicative measure) increased toward the south, with the South Island regions exhibiting higher beta diversity than the North Island regions. This pattern was observed despite our latitudinal gradient being only 1300-km long. However, Hillebrand (2004) in his review found distinct latitudinal patterns at even shorter distances, and we also observed a similar trend for both islands separately, although only for effective beta diversity. Whittaker's multiplicative beta was originally based on richness data. Nevertheless, abundance information is an important aspect of community structure, and results using species abundances can differ strongly from those obtained with richness data alone (Jost 2007). Indeed, patterns based on effective diversity were different from those for richness, likely resulting from the relatively species-poor nature of New Zealand stream invertebrate communities and the large proportion of generalist species (Collier and Winterbourn 2000). Species exhibiting high occupancies (>80% of sites) were equally common on both islands, thus reducing effective beta diversity differences because this approach places more weight on abundant species (Jost 2007). In contrast, species that exhibit low occupancies, and therefore contributed strongly to richness-based measures of beta diversity, were more frequent in the South Island regions.

To date, most studies of beta diversity have failed to incorporate statistical null models, despite the known interrelationships between alpha, beta, and gamma diversity. Following the null model approach of Kraft et al. (2011), we were able to show that the observed pattern differed from the null expectation, beta deviation being still higher in the South Island catchments. Clearly, variation in the size of the regional species pool (gamma diversity) is not solely responsible for the geographical distribution of beta diversity in New Zealand streams. Instead, the environmental setting in the South Island catchments influences macroinvertebrate assemblages either by habitat filtering or through processes such as dispersal, colonization, and extinction, producing higher beta diversity.

The key driver of macroinvertebrate beta diversity (measured as multivariate dispersion, *β*_*d*_) was habitat heterogeneity. Habitat heterogeneity is a major structuring agent of biotic communities in several systems. Stegen et al. (2013) found habitat heterogeneity to be positively related to species turnover, even after controlling for the sampling effect of gamma diversity and the effect of other explanatory variables. Correspondingly, habitat and climate heterogeneity was the key factors maintaining the beta diversity of forest birds (Veech and Crist 2007). The South Island regions had higher regional heterogeneity of in-stream habitat conditions such as depth, substrate size, current velocity, slope, and canopy cover. This heterogeneity produced broader habitat differences between streams and thus higher beta diversity (“numbers of different community types within a region”, sensu Jost 2007). The relationship between beta diversity and topographic steepness showed a similar trend, indicating that higher beta diversity could be partly a product of greater among-stream dispersal limitation in the South Island streams with steeper catchments, although this relationship was nonsignificant. Finn et al. (2006) found that the genetic diversity of an alpine stream macroinvertebrate was related to catchment steepness, suggesting dispersal limitation even at very small spatial extents. Astorga et al. (2012) showed that macroinvertebrate species with low dispersal ability exhibited a steep distance decay function, while more effective dispersers showed no relationship to geographic distance, thus emphasizing the key role of dispersal to spatial turnover in benthic macroinvertebrate communities (see also Thompson and Townsend 2006). Thus, dispersal limitation in very steep catchments could be partly responsible for the higher beta diversity in the South Island regions, but not to the same extent as habitat heterogeneity.

The productivity–beta diversity relationship has received little consideration in lotic systems. In ponds, the productivity–diversity relationship has been found to be scale dependent: regional richness and productivity are positively related, while the richness productivity pattern at the local scale is unimodal. Beta diversity (between-pond dissimilarity) must then increase with productivity to reconcile low local, yet high regional richness at high productivities (Chase and Leibold 2002). Chase (2010) explored experimentally the mechanism involved in the relationship between beta diversity of pond macroinvertebrates and productivity, and suggested that an increasing role for stochastic assembly processes leads to higher beta diversity with increasing productivity. Such a positive relationship between productivity and beta diversity has also been observed at much larger spatial extents, for example, plants on serpentine soils in California (Harrison et al. 2006) and butterflies in Canada (Andrew et al. 2012). Death (2002) showed benthic invertebrate species richness to increase with productivity in New Zealand forest streams, and interestingly, our data showed the same pattern at a much larger spatial extent (see Appendix S4). In our study, however, chlorophyll-a decreased southwards and beta diversity was thus negatively related to regional productivity. As a productivity surrogate, chlorophyll-a has the weakness of being negatively affected by in-stream disturbance, rendering the detection of a productivity response potentially confounded by disturbance. Indeed, the outlying data point in the beta diversity–productivity relationship, the Tararua region, is characterized by a high frequency of rainfall-induced floods (Death and Joy 2004), a likely reason for the weak productivity signal in this catchment. Chlorophyll-a was, however, significantly correlated with potential evapotranspiration (Spearman *r* = 0.46), a frequently used catchment-scale surrogate for productivity, and controlling for in-stream disturbance (Pfankuch index) had little effect on this correlation (partial *r*_*s*_ = 0.50). These results suggest that chlorophyll-a was a useful surrogate for productivity in our data. In support of this, Death and Zimmermann (2005) showed that while chlorophyll-a in open streams is correlated with disturbance, in forest streams, it is not. Thus, the lack of a positive productivity signal does not seem to reflect the higher frequency of floods in the South Island streams, but rather a genuinely negative relationship between productivity and beta diversity in stream invertebrate communities. Correspondingly, Stegen et al. (2013) found a robust negative relationship between bird species turnover and primary productivity in North America, strongly suggesting that higher primary productivity lowers the probability of local extinction, thus reducing species turnover.

In-stream disturbance is known to be a key determinant of species richness and evenness in New Zealand streams (Death 2002). At larger scales, Vinson and Hawkins (2003) found hydrological variability, a surrogate for disturbance, to be one of the key factors linked with global patterns in stream insect richness. Given the temperate climate and plentiful rainfall in New Zealand, many streams experience frequent floods, especially in the South Island. Floods modify stream habitats and generate patchiness, with concomitant changes in the biotic composition and spatial configuration during the recovery process (Lake 2000). Thus, we expected disturbance variability to have a role in creating beta diversity patterns across regions, especially in the South Island. Although disturbance was not significant in univariate regressions, multimodel inference analysis included the Pfankuch index in 33% of the models. Thus, higher disturbance frequency, and perhaps higher among-stream variability in recovery from disturbance, may contribute to higher species turnover in the South Island.

During the last years, a large body of ecological literature has been concerned with understanding beta diversity, and its complex linkages to dispersal, species traits, productivity, habitat heterogeneity, and gamma diversity. However, only one recent study has accounted for the influence of gamma diversity on beta diversity while simultaneously examining ecological drivers of beta diversity (Stegen et al. 2013). Following the same protocol, we found an inverse latitudinal beta diversity pattern of benthic macroinvertebrates in New Zealand streams. Interestingly, and in spite of our large study extent, habitat heterogeneity (stream reach conditions) was the strongest correlate of macroinvertebrate beta diversity, suggesting that high local-scale environmental heterogeneity may be the strongest determinant of beta diversity in stream systems, more so than regional- or catchment-scale variables.

## Supporting Information

**Appendix S1.** Map of New Zealand showing the location of the 120 stream sites across the eight regions (NL: Northland, UR: Urewera, EG: Egmont, TA: Tararua, KA: Kahurangi, AP: Arthur's Pass, WE: Westland and FI: Fiordland). Fifteen streams were sampled in each region. **Appendix S2.** Environmental variables included in data analysis. **Appendix S3.** Subset of the best candidate models accounting for *β*_*d*_ diversity. **Appendix S4.** Relationship between chlorophyll-a and macroinvertebrate species richness across all study sites.
