# Supplementary material for: Habitat heterogeneity drives the geographical distribution of beta diversity: the case of New Zealand stream invertebrates
Source: Ecol Evol. 2014 Jun 2;4(13):2693–702. doi: 10.1002/ece3.1124 (PMC4113293; doi:10.1002/ece3.1124)
Supplement: Supplementary file 1 [file ece30004-2693-SD1.docx]

**S1.** Map of New Zealand showing the location of the 120 stream sites across the eight regions (NL: Northland, UR: Urewera, EG: Egmont, TA: Tararua, KA: Kahurangi, AP: Arthur’s Pass, WE: Westland and FI: Fiordland). Fifteen streams were sampled in each region.

**S2**. Environmental variables included in data analyses.

| ***Name of variable*** | ***Units*** | ***Description*** | | |  |
| --- | --- | --- | --- | --- | --- |
| Stream segment slope | cm/m | Measured slope | | |  |
| Depth | m | Mean stream depth | | |  |
| Current velocity | m/s | Mean current velocity | | |  |
| Canopy cover | % | Mean canopy cover | | |  |
| Chlorophyll-a | µg/cm^2^ | Measured from stream stones | | |  |
| Bryophyte cover | % | Percentage cover of stream bryophytes | | |  |
| Bottom Pfankuch | dimensionless | Bed stability | | |  |
| Substrate size | dimensionless* | Measured in situ | | |  |
| Catchment steepness | %/100 | Proportion of catchment with steep slope (>30°) | | |  |
| *modified Wentworth scale (from 1 to 13, see text for further detail). | | |  |  | |
|  |  |  | | |  |
|  |  |  | | |  |
|  |  |  | | |  |
|  |  |  | | |  |
|  |  |  | | |  |
|  |  |  | | |  |

**S3.** Subset of the best candidate models accounting for β_d_ diversity. The subsets were selected based on delta AIC < 2 (difference between the AIC value of the best model and of the other models presented). The environmental variables included in the global model were: iH: in-stream heterogeneity; chla: chlorophyll-a, cS: catchment steepness; bP: Pfankuch index. *AICc* depicts AIC corrected for small sample size, *weight* is the model weight (the probability that a given model is the best approximating model).

|  | **Candidate models** | **AIC_c_** | **delta** | **weight** |
| --- | --- | --- | --- | --- |
| 1 | iH+chla | 849.2 | 0.00 | 0.354 |
| 2 | iH | 850.2 | 0.98 | 0.217 |
| 3 | iH+chla+bP | 850.9 | 1.56 | 0.153 |
| 4 | iH+chla+cS | 851.0 | 1.67 | 0.144 |
| 5 | iH+bP | 851.2 | 1.80 | 0.132 |

**S4**. Relationship between chlorophyll-a and macroinvertebrate species richness across all study sites. Fitted line is loess (local polynomial regression fitting, span=0.75).
